# FUE for the repair of unnaturally designed or low hairlines in men with androgenetic alopecia: a retrospective, multicenter observational study

**DOI:** 10.3389/fmed.2026.1735251

**Published:** 2026-03-12

**Authors:** A. Gómez-Zubiaur, J. M. Mir-Bonafé, O. Muñoz Moreno-Arrones, A. Burgos, C. de Hoyos, S. Vañó-Galván, P. Martín-Carrasco, M. Mir-Bonafé, D. Saceda-Corralo, J. M. Ricart-Vayá

**Affiliations:** 1 Trichology Unit, Ricart Medical Institute, Madrid, Spain; 2 TricologiaMir—Hair Transplant Unit, Palma de Mallorca, Spain; 3 Capilderm, Madrid, Spain; 4 Dr. Antonio Burgos Clinic, Málaga, Spain; 5 CETA Clinic, Madrid, Spain; 6 Hair Disorders Unit, Grupo Pedro Jaén, Madrid, Spain; 7 Trichology Unit, Dermavit Salud, Sevilla, Spain

**Keywords:** correction, design, extraction, hair transplant, hairline, punch, satisfaction

## Abstract

Hairline reconstruction in men with androgenetic alopecia is one of the most frequently requested and technically challenging procedures. Patients are often dissatisfied with designs that are too aggressive, straight, or rounded and may seek corrective surgery, which requires specialized planning and execution. We describe our approach using follicular unit excision to repair unsatisfactory hairline designs. A retrospective, multicenter observational study was conducted. A total of 20 men with a median age of 37.5 years who underwent follicular unit excision (FUE) repair of unnatural hairlines were included in the study. A serrated punch was used in 68.4% of cases, with a median size of 0.8 mm, and a single rotation movement of the extraction device was used in 70% of cases. The rates of total and partial transection were high. Approximately four out of five patients showed no visible scarring or pigmentary alteration at 6–12 months, and only 28% required a second corrective procedure. Patient-reported outcomes were highly favorable, with 95% expressing satisfaction or high satisfaction. FUE is a valuable and versatile technique for correcting poorly designed or excessively low hairlines. These results not only demonstrate the technical difficulty of these cases but also highlight that the majority of them can be successfully corrected with a single intervention. Corrective FUE is a practical and effective option for repairing an inappropriate hairline design when performed with meticulous attention to technical nuance.

## Introduction

The number of hair transplant surgeries has increased significantly in recent years. Hairline reconstruction is one of the most frequently requested procedures for men with androgenetic alopecia (AGA) and one of the most technically challenging for the surgeon. A well-designed frontal hairline requires careful attention to density, shape, and the gradual transition from fine to thick hair to achieve a natural and satisfactory result (1). The number of second procedures to repair previous surgery has also increased substantially. While minor corrections, such as density improvement or refinement with single follicular units, are relatively straightforward, modifying an excessively low or poorly designed hairline (such as a feminine hairline) remains a challenging task. Patients often present after undergoing a previous procedure in centers where design principles were not adequately respected, and the corrective procedure requires specialized planning and execution (1, 2).

We describe our approach using follicular unit excision (FUE) to repair unsatisfactory hairline designs, highlighting technical details that facilitate safe extraction and optimize esthetic results (3).

## Method

A retrospective, multicenter observational study was conducted. Some leading Spanish hair transplant centers were contacted, and ultimately, a total of 7 centers and 10 hair transplant surgeons participated in the study. Each hair transplant surgeon included between 1 and 3 patients. Three of the participating clinics had two hair transplant surgeons who included patients. We included men with AGA who were dissatisfied with a previous hairline design, typically because it was excessively low, rounded, or lacked a natural transition zone, and who underwent hairline repair using the FUE extraction technique. All patients were followed for at least 12 months and signed informed consent. Images of the procedure and follow-up were systematically obtained in accordance with the protocol of each participating clinic. The surgeons were asked to evaluate these images at 3, 6, and 12 months after the procedure and classify them into the following categories: no scarring or pigmentation changes, hyperpigmented punctate lesions, hypopigmented punctate lesions, erythematous punctate lesions, hypertrophic punctate lesions, and depressed punctate lesions. It was necessary to have standardized images taken at the same distance and under the same conditions during the evaluation period. The surgeons were also asked to rate their level of satisfaction with the outcome for each patient using the following scale: very satisfied, satisfied, indifferent, dissatisfied, and very dissatisfied. In addition, they completed a questionnaire on the variables of the surgical technique used in each patient, including the type and size of punch, the movement of the extraction motor, and the destination of the extracted follicular units (all response options were provided via a drop-down menu in Excel to avoid unanalyzed free-text fields). Finally, they were asked to report their usual total and partial transection rates, as well as those obtained in the cases included in the study, distributed in intervals. The study was conducted in accordance with the Declaration of Helsinki. Statistical analysis was performed using IBM SPSS^®^ (Statistical Package for the Social Sciences) version 27, EEUU. Quantitative variables were expressed as median and range, and qualitative variables were expressed as percentages of valid cases.

## Results

**Figure 1:**
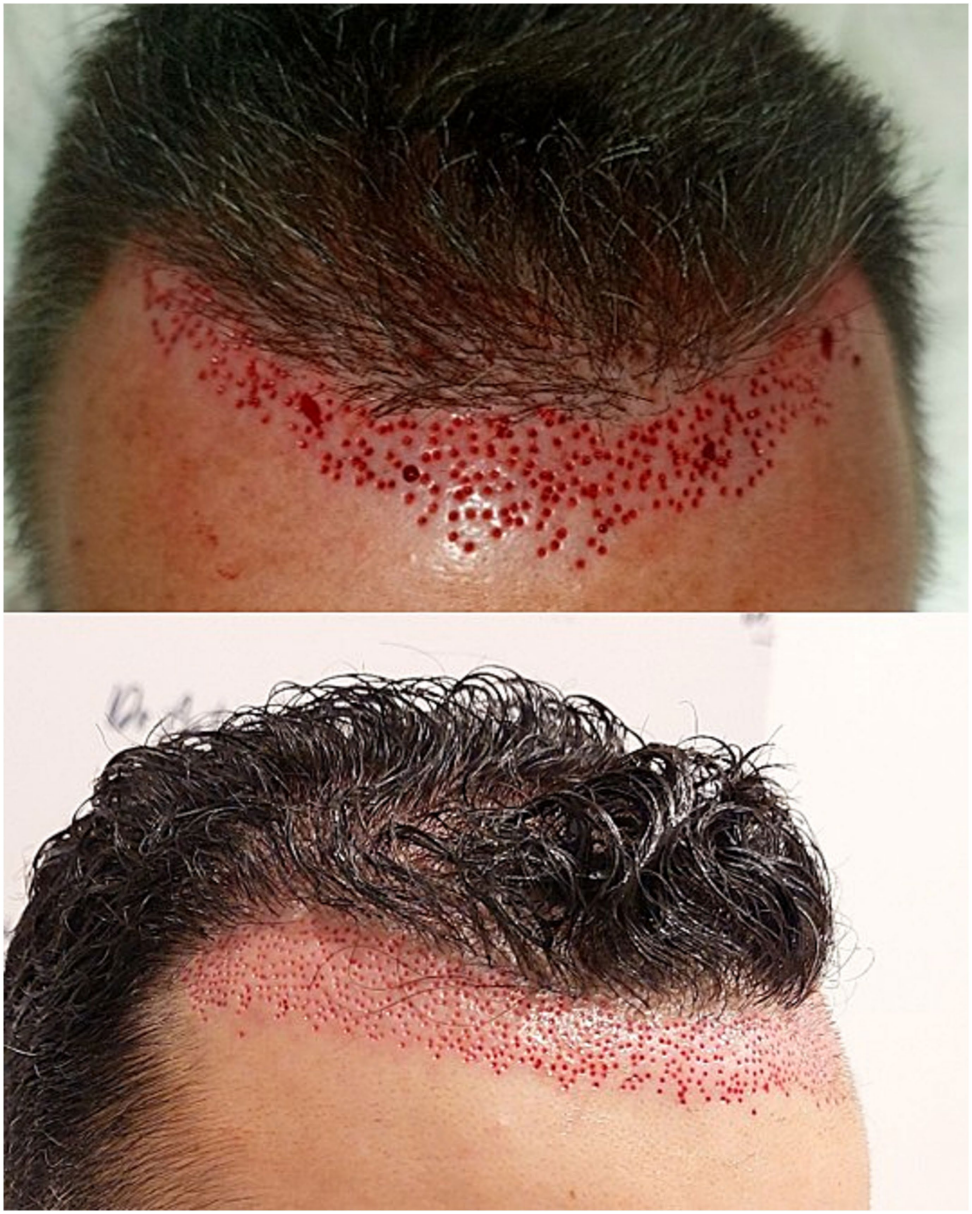
Extraction of unwanted follicular units using the FUE technique with a 0.7 mm serrated punch from two of the patients included in the series.

**Figure 2:**
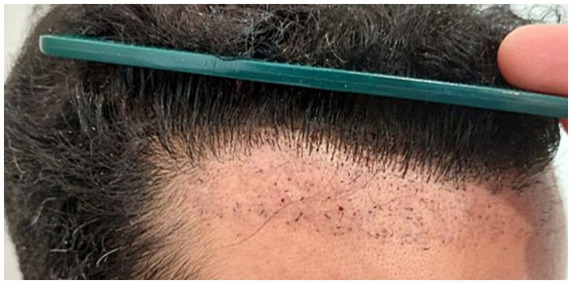
Pink punctate lesions at 3 months after FUE extraction in one of the patients included in the series. Transected follicular units are also observed in the extraction and subsequent regrowth.

**Figure 3:**
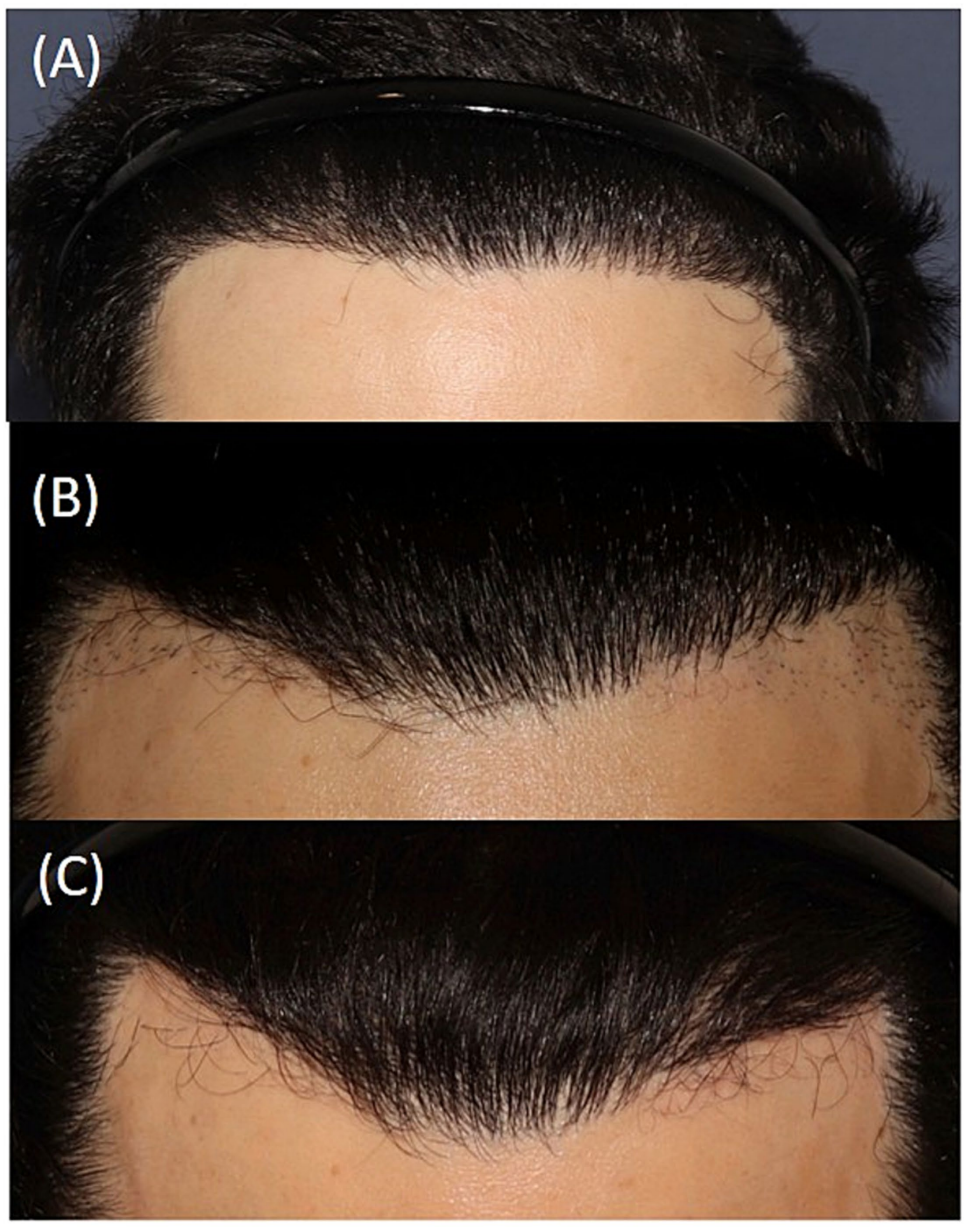
Sequential images of another patient who underwent surgery. **(A)** Prior to corrective FUE extraction. **(B)** Result 1 year after surgery, with the presence of the remaining follicular units that were not extracted and underwent a new extraction. **(C)** Result 1 year after **(B)**, with skin without lesions and without remaining follicles.

In our retrospective, multicenter study on FUE repair of unnatural hairlines, a total of 20 men with a median age of 37.5 years (range: 29–56 years) and a diagnosis of AGA were included (50% phototype II, 35% phototype III, phototype 10% IV, and 5% phototype I). Regarding the surgical technique, a serrated punch was used in 68.4% of cases, a smooth punch in 10.5%, a blunt punch in 10.5%, and a flared punch in the remaining 10.5%, with a median size of 0.8 mm (range: 0.6–0.9 mm). A single rotational movement of the extraction device was used in 70% of cases; in the remaining 30%, rotation and oscillation were combined during extraction (Figure 1). In 57.9% of cases, tumescence was not used. In 60% of cases, the extracted follicular units were implanted in the adjacent region; in 20%, they were placed in other areas; and in the remaining 20%, they were discarded (patients did not want to place them in other areas of hair loss) (Table 1). Table 2 shows the frequencies of partial and total transection rates in patients who underwent surgery, categorized by severity range. Three months after the procedure, 40% of patients had erythematous punctate lesions in the extracted area (Figure 2), whereas at 6 and 12 months after the procedure, approximately 4 out of 5 patients showed no visible scarring or pigmentary alteration (Figure 3). Only 27.8% of patients required a second procedure; in the remaining 72.2% of patients, no further FUE extraction surgery was necessary. Patient-reported outcomes were highly favorable, with 95% expressing satisfaction or high satisfaction.

**Table 1:** Surgical technique characteristics and management of follicular units.

Surgical technique characteristics	
Type of punch used	Serrated: 68.4% Smooth: 10.5% Blunt: 10.5% Flared: 10.5%
Punch size (mm)	Median: 0.8 mm Range: 0.6–0.9 mm
Extraction device movement	Single rotational movement: 70% Combined rotation and oscillation: 30%
Use of tumescence	Not used: 57.9% Used: 42.1%
Management of follicular units	
Destination of the extracted follicular units	Implanted in adjacent regions: 60% Implanted in different areas: 20% Discarded: 20%

**Table 2:** Partial (one or more stems of the follicle but not all of them) and total (all follicle stems) transection rates during punch extraction of follicular units in the study.

Transection rate intervals	Partial transection rate	Total transection rate
None	5.3%	10%
1–5% (very low)	10.5%	30%
6–10% (low)	26.3%	**45%**
11–15% (intermediate)	21.1%	10%
16–20% (high)	**31.6%**	5%
More than 20% (very high)	5.3%	0%

It should be noted that partial transection rates were high in 31.6% of the cases, and total transection rates were low in 40% of the cases. These figures will be compared with those typically reported by participating hair transplant surgeons and will give us an idea of the complexity of the surgical technique. Bolded values are the highest percentages.

## Discussion

The hair surgeon must advise the patient on the hairline design, taking into account their ethnicity, the characteristics of their alopecia, and anatomical reference points (3, 4). We propose the extraction of follicular units using the FUE technique as an alternative for repair, achieving good esthetic results and without visible scars. It should be noted that this is a more complex procedure, as the follicular units have been previously implanted in different directions and have undergone a process of healing and subsequent fibrosis.

Choosing the appropriate punch is critical. We recommend using the smallest possible diameter that allows safe extraction without causing excessive partial transection. Small punches (approximately 0.7–0.8 mm) reduce the risk of visible scarring, which is particularly important in frontal areas where previously implanted grafts must be removed. However, if the punch is too small relative to the follicular unit size, there is an increased risk of incomplete dissection and transection. Therefore, the surgeon must balance minimizing punch diameter while still ensuring an atraumatic harvest.

In the majority of cases, we prefer serrated punches, which provide better cutting efficiency and allow cleaner separation of the follicular unit from the surrounding fibrous tissue. However, in more complex cases—particularly those with dense fibrosis or follicles implanted at unusual angles—we recommend trumpet-shaped (flared) punches. These punches increase the safety margin around the follicle, improving yield and reducing transection in challenging tissue, although they result in slightly larger wounds.

Extraction is typically performed with monophasic motor devices, using a single rotational movement to penetrate the epidermis and dermis. This method is effective and sufficient in the majority of standard cases. However, in fibrotic tissue or when follicular angulation is altered, which is common in repair procedures, monophasic rotation may lead to higher transection rates. In our study, primarily using monophasic motor devices, only 28% of cases required a second corrective procedure, reflecting the technical difficulty of these cases while also demonstrating that the majority could be successfully addressed with a single intervention. Partial and total transection rates (partial: 31.6% in the high interval 16–20%; total: 45% in the low interval 6–10%) were higher than usual for the participating surgeons (usual transection rates: partial in the low interval 6–10%; total in the very low interval 1–5%).

The need for a second surgical procedure is closely related to total and partial transection rates, as the objective of the technique is to remove unwanted follicular units from their location. Units that are totally or partially transected may grow back in the same location, either fully or with some of their stems. If the number of such follicular units is high, the cosmetic result may be unsatisfactory, and the patient may want a new procedure to “clean up” the remaining follicles.

For these challenging situations, we prefer multiphasic devices, which allow the movement sequence to be tailored. A common strategy is to use rotation to pierce the epidermis, followed by oscillation to dissect the dermis and surrounding fibrotic tissue (5). This combination reduces torsion and mechanical stress on the follicular unit, thereby improving graft quality and minimizing the risk of damage. Multiphasic systems provide greater control, particularly when follicles are scarred, irregularly angled, or lying flat against the dermis.

Future studies should clarify the role of tumescence, which some surgeons avoid to prevent distortion of follicular orientation, but which may, in certain cases, improve visualization and extraction safety.

Once extracted, follicular units are evaluated for viability. In many cases, units are reimplanted in adjacent areas to enhance density or refine transitions. When design corrections are required in more distant areas, grafts can be relocated accordingly. In cases where the unit quality is compromised or the follicle is unnecessary for correction, grafts are discarded. This selective approach maximizes esthetic outcomes while avoiding unnecessary placement of low-quality grafts.

Patients are instructed in standard postoperative wound care, including gentle cleansing and avoidance of trauma. At approximately 3 months, erythematous punctate lesions may be observed in the donor area, reflecting the healing process. Importantly, by 6 to 12 months, the vast majority of patients demonstrate complete resolution of these findings, with no visible scarring or pigmentation changes in 79% of cases.

In addition, non-surgical options such as laser hair removal and hair electrolysis may provide alternative strategies for eliminating undesired follicular units in selected patients. However, laser hair removal carries a known risk of paradoxical hypertrichosis in adjacent areas when performed on the face, and electrolysis typically reduces hair diameter rather than achieving complete removal of the hair shaft, often requiring multiple sequential sessions to reach the desired outcome (6).

The limitations of the study include the small sample size and those arising from its retrospective design. Although image acquisition was standardized, it differed between centers, and no single evaluator was designated. In addition, while all procedures used FUE, the surgical technique varied across centers. Finally, no validated scale was employed to assess patient satisfaction.

FUE is a valuable and versatile technique for correcting poorly designed or excessively low hairlines. By carefully selecting punch type and diameter, adapting motor settings to tissue conditions, and tailoring graft management, surgeons can achieve natural, nearly scar-free results with high patient satisfaction. Surgeons must contend with previously implanted follicles oriented in diverse directions and surrounded by varying degrees of scar tissue and fibrosis. This unpredictability requires meticulous planning and adaptability. Although the retrospective nature of our series and the limited sample size are notable limitations, our experience suggests that corrective FUE is a practical and effective option when performed with careful attention to technical detail.

Key technical pearls:

- Use the smallest punch diameter possible, while avoiding an increase in transection rates.
- Serrated punches are effective in most scenarios, whereas trumpet-shaped punches may be advantageous in dense fibrosis or extreme angulation.
- Monophasic motor rotation is adequate for standard corrections, but multiphasic motion (rotation + oscillation) improves outcomes in complex, fibrotic cases.
- Maintain transection rates within reference ranges (partial: 6–10%; total: 1–5%), ensuring graft viability and minimizing unnecessary tissue trauma.

## Data Availability

The original contributions presented in the study are included in the article/supplementary material, further inquiries can be directed to the corresponding author.
